# Free-Ranging Male Koalas Use Size-Related Variation in Formant Frequencies to Assess Rival Males

**DOI:** 10.1371/journal.pone.0070279

**Published:** 2013-07-29

**Authors:** Benjamin D. Charlton, Desley A. Whisson, David Reby

**Affiliations:** 1 School of Psychology, University of Sussex, Brighton, United Kingdom; 2 Centre for Integrative Ecology, School of Life and Environmental Sciences, Deakin University, Burwood, Victoria, Australia; University of Tours, France

## Abstract

Although the use of formant frequencies in nonhuman animal vocal communication systems has received considerable recent interest, only a few studies have examined the importance of these acoustic cues to body size during intra-sexual competition between males. Here we used playback experiments to present free-ranging male koalas with re-synthesised bellow vocalisations in which the formants were shifted to simulate either a large or a small adult male. We found that male looking responses did not differ according to the size variant condition played back. In contrast, male koalas produced longer bellows and spent more time bellowing when they were presented with playbacks simulating larger rivals. In addition, males were significantly slower to respond to this class of playback stimuli than they were to bellows simulating small males. Our results indicate that male koalas invest more effort into their vocal responses when they are presented with bellows that have lower formants indicative of larger rivals, but also show that males are slower to engage in vocal exchanges with larger males that represent more dangerous rivals. By demonstrating that male koalas use formants to assess rivals during the breeding season we have provided evidence that male-male competition constitutes an important selection pressure for broadcasting and attending to size-related formant information in this species. Further empirical studies should investigate the extent to which the use of formants during intra-sexual competition is widespread throughout mammals.

## Introduction

Recent research on nonhuman mammal vocal communication has focused on the importance of acoustic cues that are directly linked to the caller’s phenotype and cannot easily be faked [for overview see 1]. In particular, because body size is a key determinant of competitive ability in mammals [2-4] numerous studies have investigated whether the acoustic features of mammal calls have the potential to signal reliable information about the caller’s body size. Whereas the fundamental frequency (pitch) of mammal calls is often an unreliable cue to body size [4-10], a number of studies have shown that lower and more closely spaced formants – the natural resonance frequencies of air in the vocal tract – are indicative of longer vocal tracts and larger body sizes [4,6,8,9,11-15] and also demonstrated that receivers attend to size-related formant variation during sexual or social interactions [16-26]. These observations suggest that formants could be used in reproductive contexts as cues to body size to deter rivals and/or attract females in a wide range of nonhuman mammals. Notwithstanding this, confirmation that males use formants to assess rivals in reproductive contexts is limited to two eutherian mammal species [red deer, *Cervus* *elaphus* : 18, and Australian sea lions, *Neophoca* *cinerea* : 26]. In this study, we investigated whether a metatherian mammal, the koala ( *Phascolarctos* *cinereus* ), uses formants as assessment cues during intra-sexual competition.

Koalas are marsupial mammals that inhabit the *Eucalyptus* forests of eastern Australia [27]. The koala’s solitary nature and conspicuous vocal activity during the breeding season [28-30] indicates that vocal communication is likely to be important for coordinating this species’ reproductive behaviour, and makes the koala well suited for studying the function of vocal signals in mammal sexual communication. Recent work has revealed that the formant frequency spacing of male koala bellows is a reliable acoustic cue to the caller’s body size [6]. In addition, playback studies, using re-synthesis techniques to shift formants in male bellows, have shown that oestrous female koalas move preferentially towards male bellows with lower formants simulating larger callers [25], and also confirmed that male koalas perceive formant shifts in male bellows corresponding to the natural variation in body size between a large and small adult male [31]. Taken together, these findings indicate that inter-sexual selection pressures will favour individuals able to produce lower formants in their bellows, and also suggest that size-related formant information could be functionally relevant to male koalas.

Nevertheless, because these previous studies were conducted on captive animals, the response of koalas to size-related formant information in their natural environment remains to be investigated. Indeed, whereas captive male koalas do not display differential looking responses to male bellows simulating different size callers [31], other behavioural responses to size-related formant information (such as differences in vocal behaviour) may become apparent in free-ranging males that are actively competing for females during the breeding season. Signalling body size is important for determining the outcome of agonistic interactions between males in several terrestrial vertebrates [e.g. 18,32,33] and the male koala’s permanently descended larynx certainly suggests strong selection pressures for callers to elongate their vocal tracts and lower formants [6], in order to maximise the acoustic impression of their body size conveyed to receivers [34]. Thus, because formants are salient to male koalas [31] it is reasonable to predict that they use these acoustic cues to assess the body size of prospective rivals, allowing them to avoid escalating contests with larger, and potentially more dangerous individuals.

In the current study, we used playback experiments to examine the behavioural responses of male koalas in their natural environment to re-synthesised bellows simulating large versus small males. We hypothesise that males will be more attentive to playbacks of bellows with lower formants simulating larger males, and also slower to bellow in response to these playbacks that represent more dangerous rivals. In line with observations in red deer [18] we also expect that male koalas will invest more effort into vocal responses when presented with formants simulating larger males: specifically, we expect males to produce longer bellows with lower formants and spend more time bellowing in response to playbacks simulating larger rivals. Our results will allow us to determine whether male-male competition has provided an additional selection pressure alongside female mating preferences for broadcasting size-related formant information in this species.

## Materials and Methods

### Ethical statement

This work follows the Association for the study of Animal Behaviour/Animal Behaviour Society guidelines for the use of animals in research, and was approved by the Animal Ethics Committees at Deakin University (A01-2011) and the University of Sussex (ERC/34/E-CIRC/CHA). The study was carried out under scientific permit #10005379 from the Victorian Department of Sustainability and Environment.

### Study site and animals

The playback experiments were conducted on a free-ranging koala population at Cape Otway, Victoria, Australia, during the 2012 breeding season (Sep–Nov). The koala population at Cape Otway remains active throughout the day during the breeding season (Whisson et al, unpublished data). A total of 12 adult male koalas served as subjects in the playback experiments. Although the precise age of these free-ranging animals was not known, all the male koalas in the study had active sternal scent glands during the playback experiments, which confirms their sexual maturity and adult status [35]. Individual males were identified using a combination of very-high-frequency (VHF) radio collars and ear tags that had previously been fitted between April 2011 and September 2012 as part of a three-year study of koala habitat use and movements (Whisson et al, unpublished data). In this study, koalas were captured using a standard ‘noose and pole’ technique [36]. Each animal was then restrained in a hessian bag (without anaesthesia), ear-tagged with a coloured swivel tag (10 mm x 40 mm, Leader Products Pty Ltd., Victoria, Australia), and fitted with a VHF radio collar (Sirtrack, Havelock North, New Zealand). Previous long-term studies of koala spatial movements have used radio-collars without observing any negative impact on the behaviour of study animals [e.g. 30,37]. In the current study, the collar weight of 66 g was less than 1% of the weight of the male koalas in the study population (mean = 12.3 kg; minimum = 11.2 kg).

### Selection of bellows for re-synthesis

Three bellows of comparable duration from each of eight adult male koalas aged between 3–11 years were selected for use as playback stimuli. These males were recorded at Lone Pine Koala Sanctuary, Brisbane, Australia, using a Sennheiser ME67 directional microphone and a Zoom H4N portable solid-state digital recorder (sampling rate: 44.1 kHz, amplitude resolution: 16 bits) at distances ranging between 2 and 10 metres. The free-ranging koalas in the current study were thus unfamiliar with the individuals that served as exemplars in our experiments. The recorded vocalisations were transferred onto an Apple Macintosh MacBook Pro computer, and saved in WAV format at 16 bits amplitude resolution, 44.1 kHz sampling rate.

### Acoustic analysis of playback stimuli

**Figure 1:**
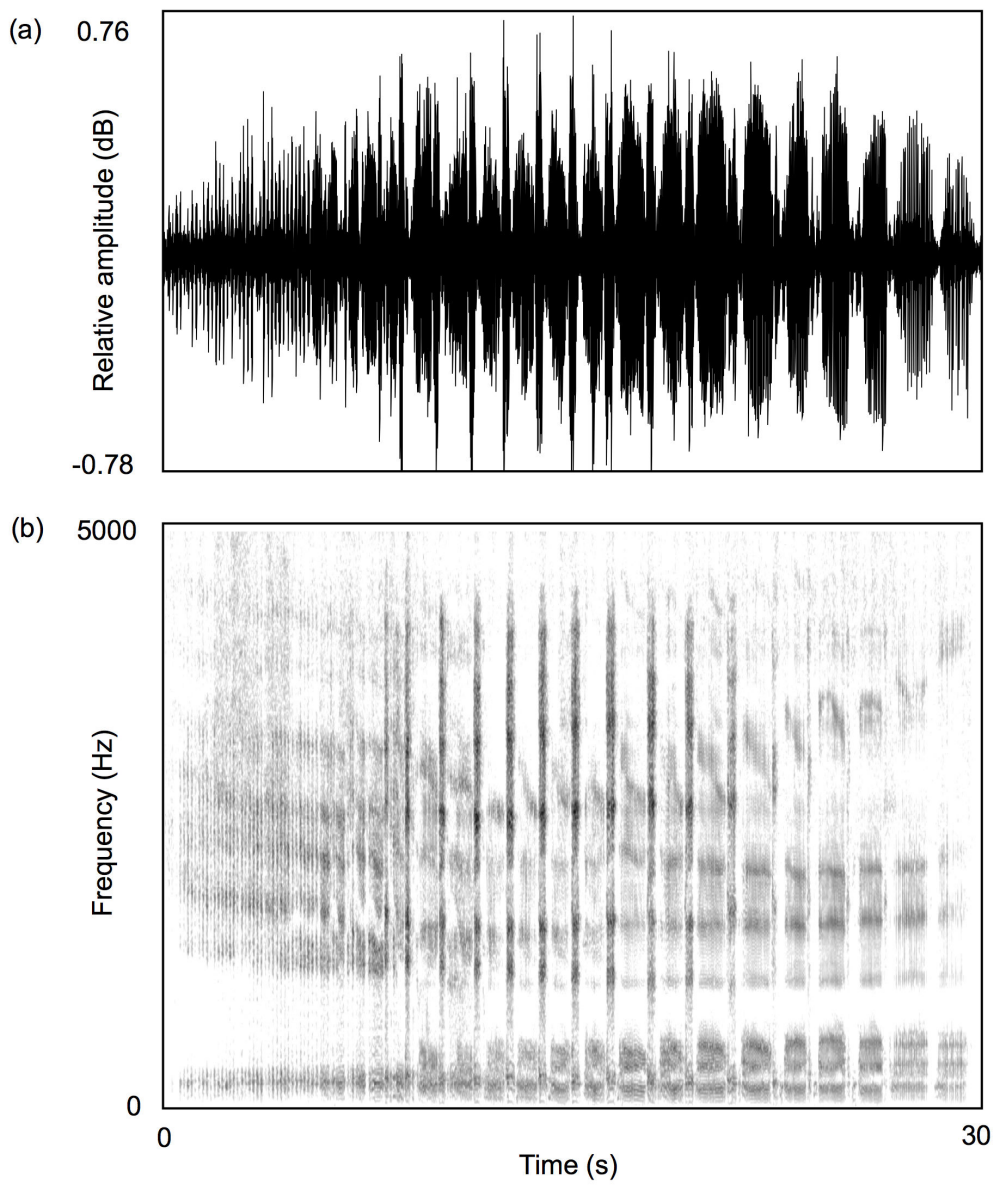
Male koala bellow. Waveform (a) and spectrogram (b) are shown (spectrogram settings: FFT method; window length 0.09 s; time step = 0.002 s; frequency step = 20 Hz; Gaussian window shape; dynamic range = 45 dB). Male bellows are characterized by an introductory phase that is followed by a continuous series of inhalations and shorter exhalations.

Male koala bellows consist of an onset phase of abrupt exhalations followed by a continuous alternating series of inhalation and exhalation sections (Figure 1). The formant frequency spacing of the later inhalation sections of bellows is more predictive of male body size than that of the exhalation or initial inhalation phases [6]. Consequently, to generate target values for our re-synthesis procedure we first of all extracted the original formant frequencies from the later inhalation phases of each bellow using Praat 5.1.03 DSP package, www.praat.org. The frequency values of the first six formants of the later inhalation sections of male bellows were measured using Linear Predictive Coding (LPC; ‘To Formants (Burg) command in Praat) and the following analysis parameters: time step: 0.01 seconds; window analysis: 0.03 seconds; maximum formant value: 2300 Hz; maximum number of formants: 6; pre-emphasis: 50 Hz. These analysis settings were established by previous studies [6,38]. The formant frequency values were then used to estimate the formant spacing (ΔF) of each bellow by regressing each formant value against its expected value if the vocal tract is approximated as a straight uniform tube open at one end, the mouth, and closed at the other, the glottis [this method is covered in more detail by 9].

### Calculation of re-synthesis factors

Before we could calculate the re-synthesis factors we first of all needed to establish a mean ΔF value for our study population. To do this we entered the mean head length of 160 mm measured from 24 male koalas at Cape Otway (Whisson et al, unpublished data) into a regression equation derived from 20 Queensland male koalas that describes the relationship between head length and ΔF in this species [6]. This gave us a mean ΔF of 291 Hz for our study population. To test the validity of this value we then obtained a recording from an average sized male koala at Cape Otway (with a head length of 159 mm) prior to study commencement, and found that our predicted mean ΔF value of 291 Hz corresponded almost exactly to the formant spacing of the later inhalation phases of this individual’s bellow. As a result, we are confident that a ΔF value of 291 Hz represents a medium sized adult male koala at our Cape Otway study site.

**Figure 2:**
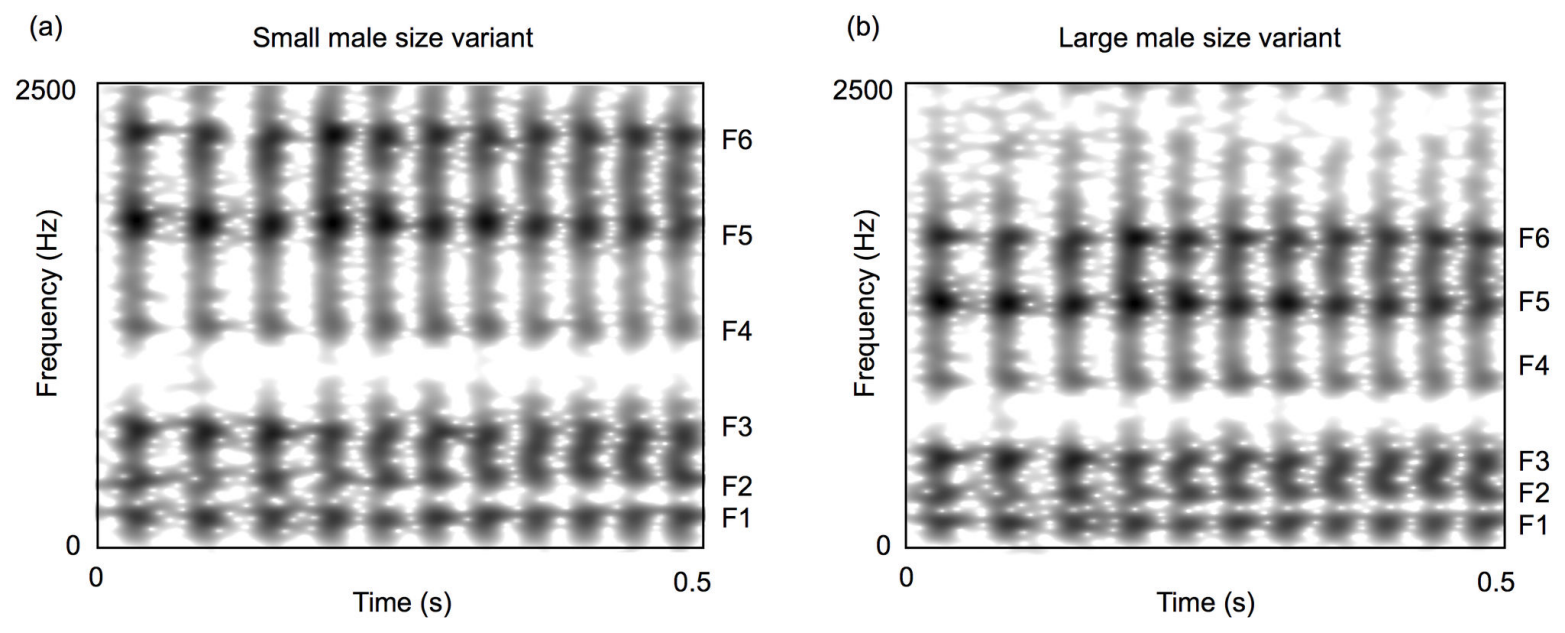
Resynthesised bellow inhalation section to illustrate the male size variants. Spectrograms of the small (a) and large (b) male size variants are shown. Spectrogram settings: FFT method; window length 0.03 s; time step = 0.002 s; frequency step = 20 Hz; Gaussian window shape; dynamic range = 35 dB. The formant frequencies are labelled F1-F6. Note that the formant frequency values and spacing are lower in the large male size variant than they are in the small male size variant.

Previous data from a population of 20 adult male koalas showed a variation of approximately 15% around the mean ΔF for the largest and smallest adult males [6]. Therefore, in order to realistically simulate natural variation in male ΔF between the largest and smallest adults representative of a population, we used values shifted ± 15% from the estimated mean of our Cape Otway population (of 291 Hz) as target ΔF values: creating large and small adult size variants with ΔF’ s of 248 and 335 Hz, respectively (Figure 2). The re-synthesis factors required to change ΔF to these values were simply calculated by dividing the intended target values, 248 or 335, by the originally measured values for each bellow.

### Re-synthesis procedure

Entire bellows were re-synthesised using a Praat script that incorporated a PSOLA (Pitch Synchronous Overlap and Add) based algorithm [39]. The script effectively speeds up or slows down the recording by a given factor (compressing or expanding the entire sound spectrum) before resetting F0 and duration to their original values, so that ΔF is changed by the required factor while leaving all other acoustic parameters unchanged [for more details see 24]. The mean intensity values of all the re-synthesised bellow stimuli were standardised to 65 dB.

### Playback sequences

The playback sequences consisted of three single bellows from each of the eight male exemplars. To simulate a realistic rate of delivery we separated each of the bellows in a sequence by five minutes (B Charlton, personal observation), making the total duration of the playback sequences between 1026 and 1095 seconds (mean = 1061 s). For each male exemplar we created a large and small size variant condition. Accordingly, each condition for a given male exemplar had exactly the same three original bellows that had been re-synthesised to represent a large or small male. In this way we controlled for all other acoustic differences between size variants. The playback sequences were burnt to CD and began with five minutes of silence.

### Playback protocol

Playback experiments were initiated when subjects were stationary and awake, and their attention was directed away from the playback speaker. Each male koala that served as a subject in our experiments was presented with stimuli in matched pairs. A pair was a subset of stimuli from the same male exemplar with formant frequencies representing either a large or a small adult. One of the stimuli in the pair was played to the subject male in the early morning and the other in the late afternoon/evening of the same day. Each subject then received the same matched pair the next day, or the day after that if poor weather prevented playback, but this time we alternated whether the stimulus representing the larger or smaller size variant was presented first. The size variant played first during the initial matched pair was also alternated across subjects. Thus, each subject received a total of 4 playback sequences: two representing a large adult and two representing a small adult.

### Playback experiments

The playback sequences were presented using a Chaiyo Focus 505 loudspeaker (Taipei, Taiwan) at sound pressure levels sounding equivalent to that of naturally bellowing males of 75 dB at 1 m from the source (determined using a Radio Shack Sound Level Meter, set for C-weighted fast response). The playback speaker was concealed in the available vegetation next to a tree that was adjacent to the subject and did not contain a male koala. The speaker was placed on a tripod, 10-15 metres from the subject (verified using Bushnell Yardage Pro laser rangefinder binoculars) and at a height of 1.5 metres from the ground. Male koalas commonly call from this height as they enter a new tree (B Charlton, personal observation). Presenting the playback stimuli at a height of 1.5 metres also allowed us to limit the effect of ground reflections [40].

Playback sequences began with five minutes of silence and to start the experiments we used a remote control device. This allowed us to limit disturbance around the speaker at playback onset. To capture vocal responses we placed a RODE NTG-2 directional microphone fitted with a foam windshield on a 1 metre high stand approximately 5 metres from the subject’s position. The microphone was attached to a Zoom H2N digital recorder set to record at a 44.1 kHz sampling rate with an amplitude resolution of 16 bits. A Sony hard drive digital camera (model DCR-SX65) was used to capture behavioural responses during the playback experimental period.

### Behavioural analysis

We used a combination of acoustic and video analyses to quantify male responses to the playback stimuli. Gamebreaker v7 digital video analysis system (SportsTec, Sydney) for Mac OS 10.6 was used to measure the duration of looks given towards the playback source. A look was defined as starting when a subject raised or turned its head to face the speaker position having previously faced away. Any movement leading to the subject looking away from the speaker defined the end of a look. We used the audio recordings captured by the microphone setup to measure the duration of bellows given in response to the playback stimuli, as well as the time taken for males to bellow after the onset of the first bellow presentation (latency to bellow). Audio and video recordings were used in conjunction to verify that the target male produced bellows recorded by the microphone setup. Bellow duration and latency to bellow were measured directly from spectrograms of the audio recordings (using the ‘Edit’ facility in Praat). In addition, the formant frequency spacing of all bellows produced in response to the playbacks (bellow formant spacing) was measured using the same approach previously described in the ‘acoustic analysis of playback stimuli’ section. To reflect the lower formants of the larger Cape Otway population the maximum formant value for the analysis was set to 1800 Hz. As before, visual inspections of spectrograms confirmed that Praat was accurately tracking the formants of bellows.

### Statistical analysis

Log (10) transformations were used to normalise the data distribution for total time spent looking, mean duration of looks, total time spent bellowing and latency to bellow. The other variables were normally distributed (Kolmogorov-Smirnov: *P* > 0.05). We used linear mixed effect models fitted with maximum likelihood estimation to examine the data. These models are particularly useful because they allow us to define both fixed and random factors. Factor effects are random if the levels of the factor used in the study represent a random sample of a larger set of potential events, for example, the subjects in our experiment and the male exemplars used to create the playback stimuli. Accordingly, because we analysed a total of 48 playbacks to 12 different subjects (24 playbacks of both large and small size variants) subject identity and male exemplar were entered as random factors, and the size condition was entered as a fixed factor in our linear mixed effect models.

Separate linear mixed effect models were computed for each of the six response variables (log (10) total time spent looking, log (10) mean duration of looks, log (10) total time spent bellowing, mean duration of bellows, log (10) latency to bellow, and bellow formant spacing) and in each case a scaled identity covariance structure was used for the repeated measures. This covariance structure proved to be the most parsimonious, having the lowest Akaike’s information criterion value of all the covariance structures in which the model reached convergence. IBM SPSS statistics version 20 for Mac OS 10.8 was used to run the linear mixed effect models, significance levels were set at *p* = 0.05, and two-tailed probability values quoted.

## Results

**Figure 3:**
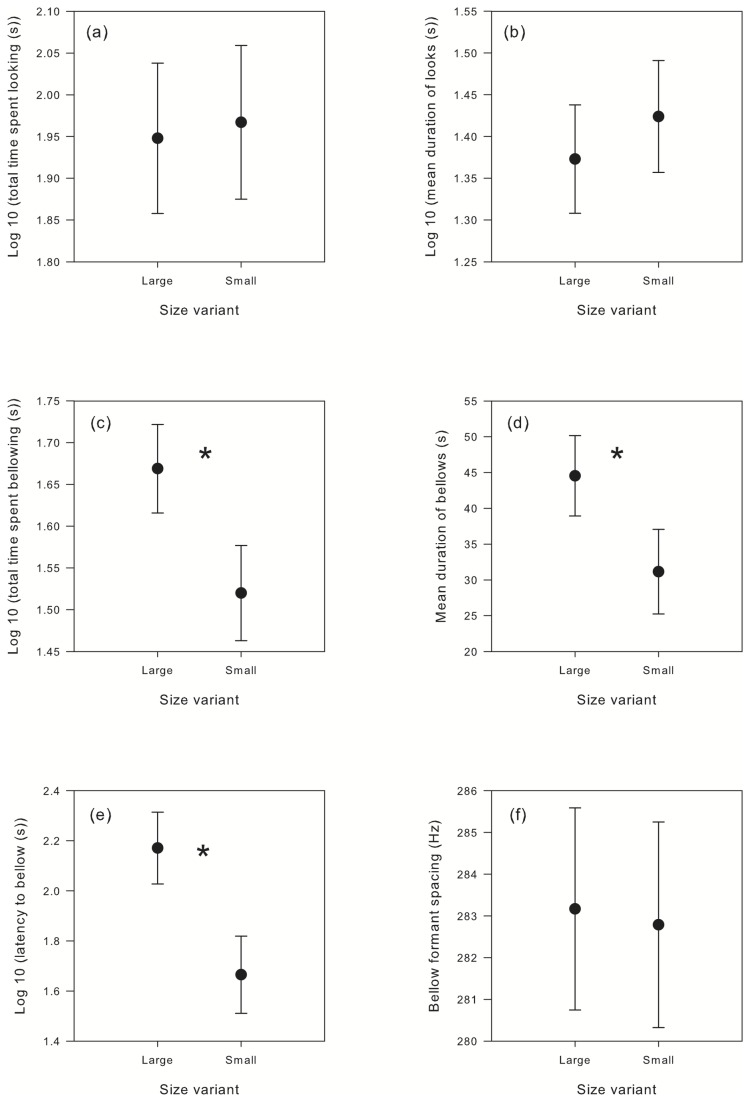
Estimated marginal means ± SE of male behavioural responses to the playback stimuli. Looking responses (a and b), bellow responses (c and d), latency to bellow (e) and bellow formant spacing (f) to the large and small adult male size variant conditions are shown. *p < 0.05.

No statistically significant difference in the total time spent looking (*F*_1,28_ = 0.022, *p* = 0.884) or the mean duration of looks (*F*_1,28_ = 0.337, *p* = 0.566) given by males in response to either size variant condition was observed (Figure 3a and b). In contrast, males spent significantly more time bellowing (*F*_1,15_ = 4.926, *p* = 0.037) and produced significantly longer bellows (*F*_1,15_ = 4.651, *p* = 0.042) in response to playbacks simulating large adults than they did in response to playbacks simulating small adults (Figure 3c and d). In addition, latency to bellow was significantly greater when males were presented with the larger size variant condition (*F*_1,15_ = 5.781, *p* = 0.023) (Figure 3e). No difference in the formant spacing of bellows produced in response to either playback condition was detected (*F*_1,15_ = 0.056, *p* = 0.815) (Figure 3f).

## Discussion

Our results show that male koalas adjust their vocal response in relation to the size-related formant information in bellows. As predicted, we found that males gave longer bellows and spent more time bellowing in response to playbacks simulating larger males. These findings indicate that male koalas use formants to assess the competitive ability of potential opponents during the breeding season, putting more effort into bellowing when faced with larger rivals that represent a greater threat. Indeed, playbacks studies on birds [41-43] frogs [44-46] and red deer [18] have all shown that males put more effort into vocal responses when presented with playbacks that simulate more threatening rivals. It is noteworthy though, that males did not lower formants in their replies to the larger size variant. These observations indicate that male koalas do not dynamically vary the size-related information in their bellows according to the perceived size of rivals, suggesting instead that males always project the maximal impression of their body size when calling.

An explanation for the longer duration of bellows given in response to playbacks simulating larger males may be found if we consider that males are attempting to further increase their apparent competitive potential during vocal interactions with larger rivals. Increased call duration could signal a highly motivated caller [47,48] and possibly one with high testosterone levels [as in giant pandas, *Ailuropoda* *melanoleuca* , 49]: and both these attributes are likely to play a deterministic role in aggressive competition among males [50,51]. Furthermore, as most vocal signals are composites of several different features they have the potential to act as multi-component signals to male quality [52,53]. For example, recent work has shown that male rock hyrax ( *Procavia* *capensis* ) ‘songs’ simultaneously encode information on body size, current condition, social status and hormonal state [54]. Accordingly, we suggest that future studies investigate whether the duration of male koala bellows has the potential to signal reliable information about the caller’s motivational state and/or testosterone levels, both of which may be functionally relevant indices of the competitive potential of callers alongside size-related information in this species’ intra-sexual communication.

We also found that male koalas were slower to reply to playbacks of bellows with lower formants, possibly because they are less willing to engage in vocal exchanges with larger males that represent more dangerous rivals. Indeed, fighting fish, *Betta* *splendens* , are slower to approach and display to the winners of previous agonistic interactions [55] and vocal communication studies on birds have also shown that males are slower to reply to playbacks of vocal signals from more dangerous opponents [32,56]. Nonetheless, males were not more attentive, as judged by looking responses, when presented with bellows simulating larger males, mirroring the findings of a previous study on captive male koalas in which mean looking duration did not differ according to the value of the formant frequency spacing in bellows [31]. It seems, therefore, that the presence of a large male in the vicinity does not represent an immediate threat, and suggests instead that agonistic interactions are mostly resolved through signalling, as they are in numerous terrestrial vertebrates [57]. This is consistent with the observation that male koalas rarely engage in direct physical confrontations for access to females [29]. In addition, recent evidence shows that the reproductive output of male koalas is correlated to their body size/weight [58]. It seems likely then, that male koalas are able to resolve the majority of agonistic interactions before they escalate into direct physical confrontations using formants to signal their apparent size. It is also conceivable that male koalas adjust their behavioural responses to bellows from different size rivals according to their own size, if they learn to associate size-related formant information with the outcomes of previous agonistic interactions. Future playback studies should test this directly by examining the response of male koalas of known body size to bellows simulating different size rivals.

In conclusion, our findings indicate that formants play an important role during vocal exchanges between male koalas, providing individuals with the means to assess the body size of rivals during the breeding season. Previous work has shown that female koalas move preferentially towards bellows with lower formants when they are in oestrus [25]. The results of the current study confirm that intra-sexual selection pressures have also played an important role in the evolution of male koala vocal displays, and add to a growing body of literature demonstrating that formants are important in the vocal communication of nonhuman mammals [16-24]. Moreover, the presence of adaptations that allow male mammals to lower formants in their sexual calls [by elongating the supra-laryngeal or nasal vocal tract: 4,34,59-61] indicates that the use of formants to broadcast information on body size is particularly important in reproductive contexts. We suggest that future work employs re-synthesis techniques and a playback approach to investigate the functional significance of formants as indicators of body size during intra-sexual competition in additional mammal vocal communication systems. Such studies may reveal that the assessment of sexual rivals using formants is widespread in nonhuman mammals.
